# Supplementary figures and images for: Fluorescent reporter lines for auxin and cytokinin signalling in barley (Hordeum vulgare)
Source: PLoS One. 2018 Apr 25;13(4):e0196086. doi: 10.1371/journal.pone.0196086 (PMC5918912; doi:10.1371/journal.pone.0196086)

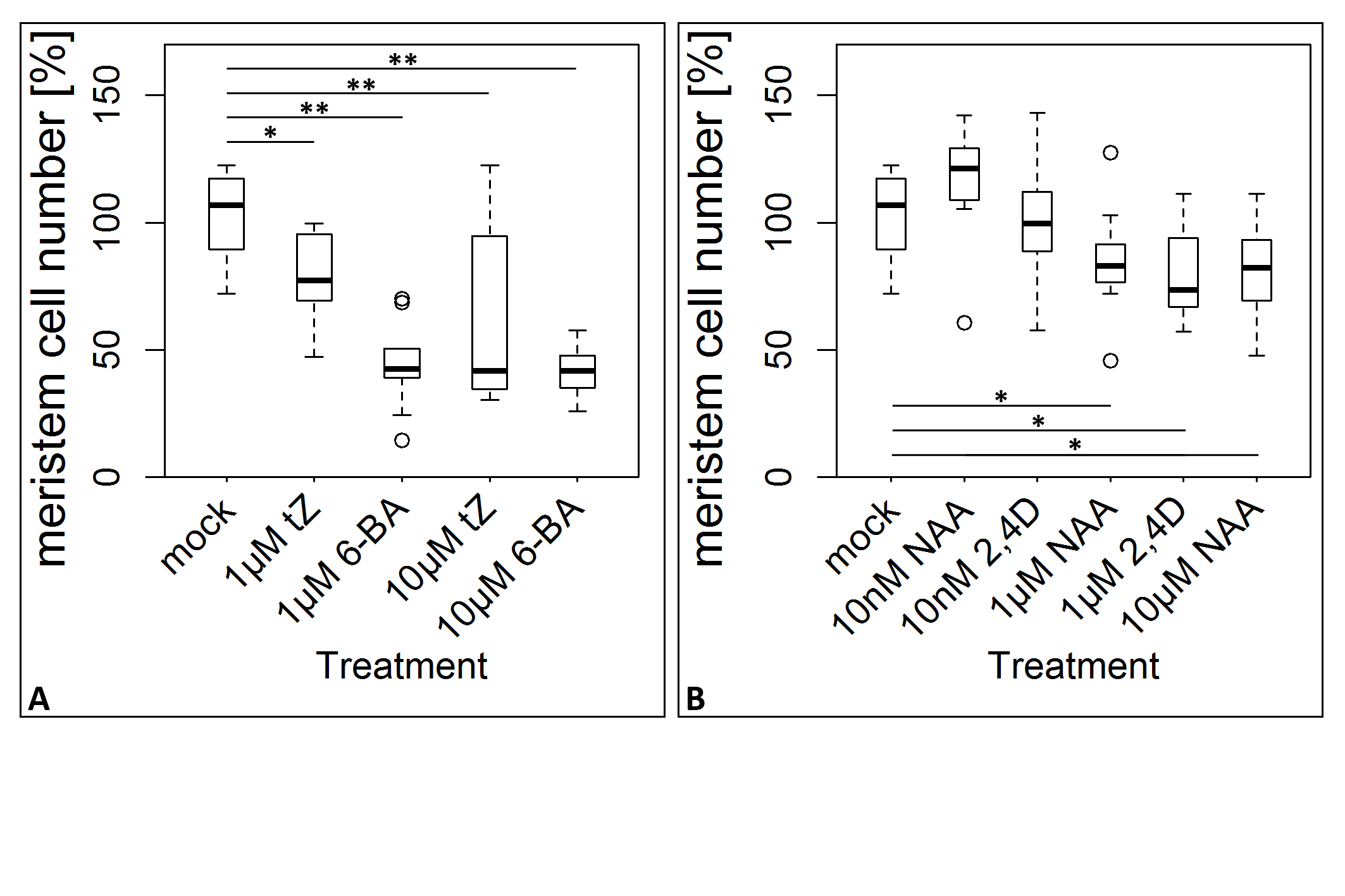

Supplement: S1 Fig — A) Meristem cell number upon 10-day cytokinin treatment; experiment was performed twice; n = 11–16 roots per data point. B) Meristem length upon auxin treatment; experiment was performed twice; all values are normalized to the mock-treated control; n = 7–17 roots per data point; significance was determined using the two-tailed Student’s t test, * = p<0.05, ** = p<0.001. (TIF) [file pone.0196086.s001.tif]

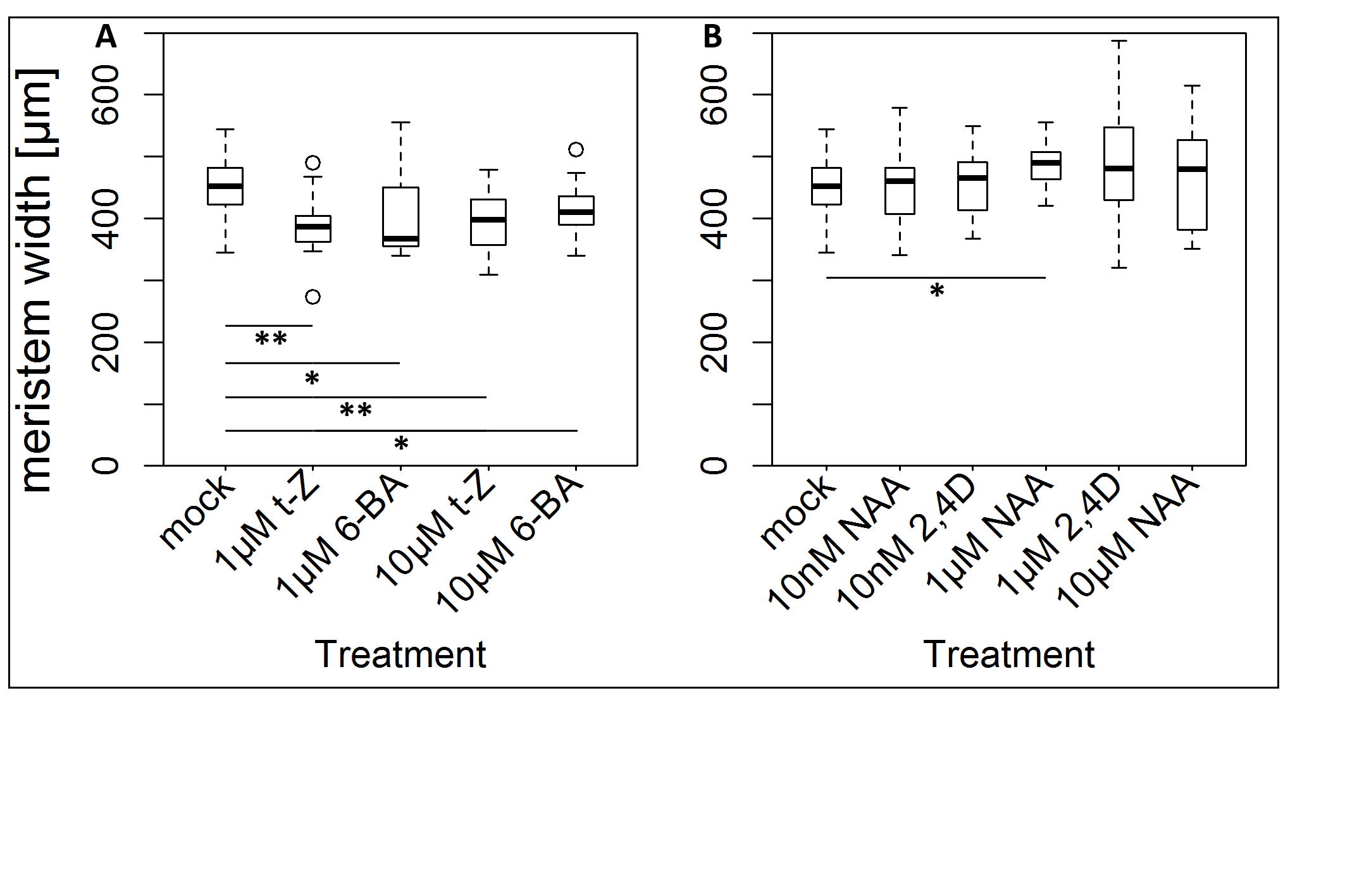

Supplement: S2 Fig — Meristem width measured at the transition zone from root meristems exemplarily shown in Figs 1B and 3B. A) Roots were treated with cytokinin for 10 days; experiments were performed twice; n = 15–25 roots per data point. B) Roots were treated with auxin for 10 days; experiment was performed twice, n = 12–25 roots per data point; significance was determined using the two-tailed Student’s t test, * = p<0.05, ** = p<0.001. (TIF) [file pone.0196086.s002.tif]

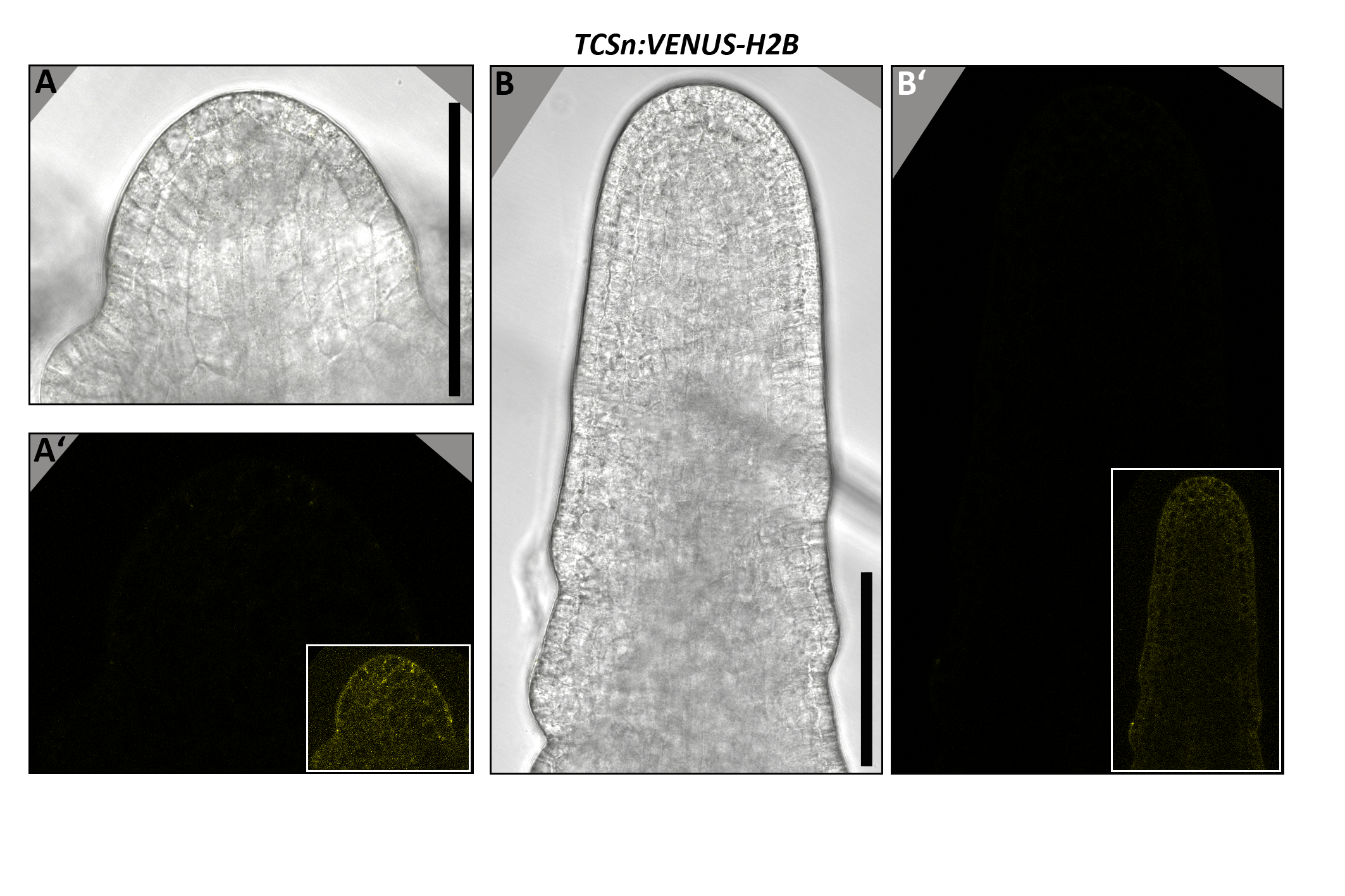

Supplement: S3 Fig — A), A’) Undectectable TCSn:VENUS-H2B expression in SAMs in waddington stage I; transmitted light and VENUS emission (A)) and VENUS emission only (A’)). B), B’) Undectectable TCSn:VENUS-H2B expression in SAMs in waddington stage II; transmitted light and VENUS emission (B)) and VENUS emission only (B’)). Seven independent transgenic lines were examined and show no expression in the SAM; scale bars 100 μm; insets in A’) and B’) show respective pictures with tonal correction to show autofluorescence. (TIF) [file pone.0196086.s003.tif]

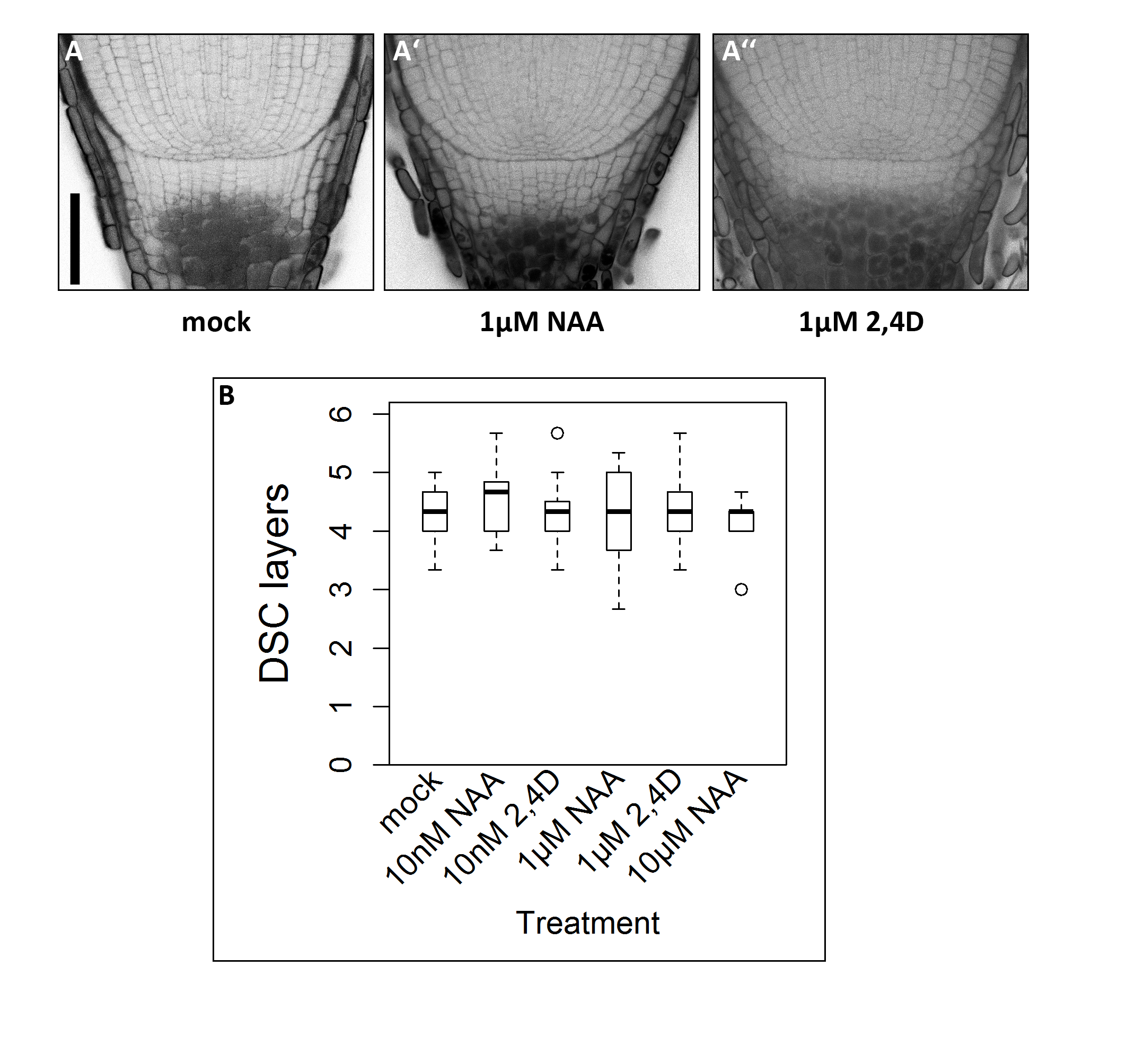

Supplement: S4 Fig — A) Exemplary pictures of the root stem cell niche upon mock or auxin treatment as indicated; scale bar 100 μm. B) Number of DSC layers upon 10-day treatment with auxin; no significant difference to mock-treated plants; experiment was performed twice; n = 5–20 per data point. Significance was determined using the two-tailed Student’s t test, * = p<0.05, ** = p<0.001. (TIF) [file pone.0196086.s004.tif]

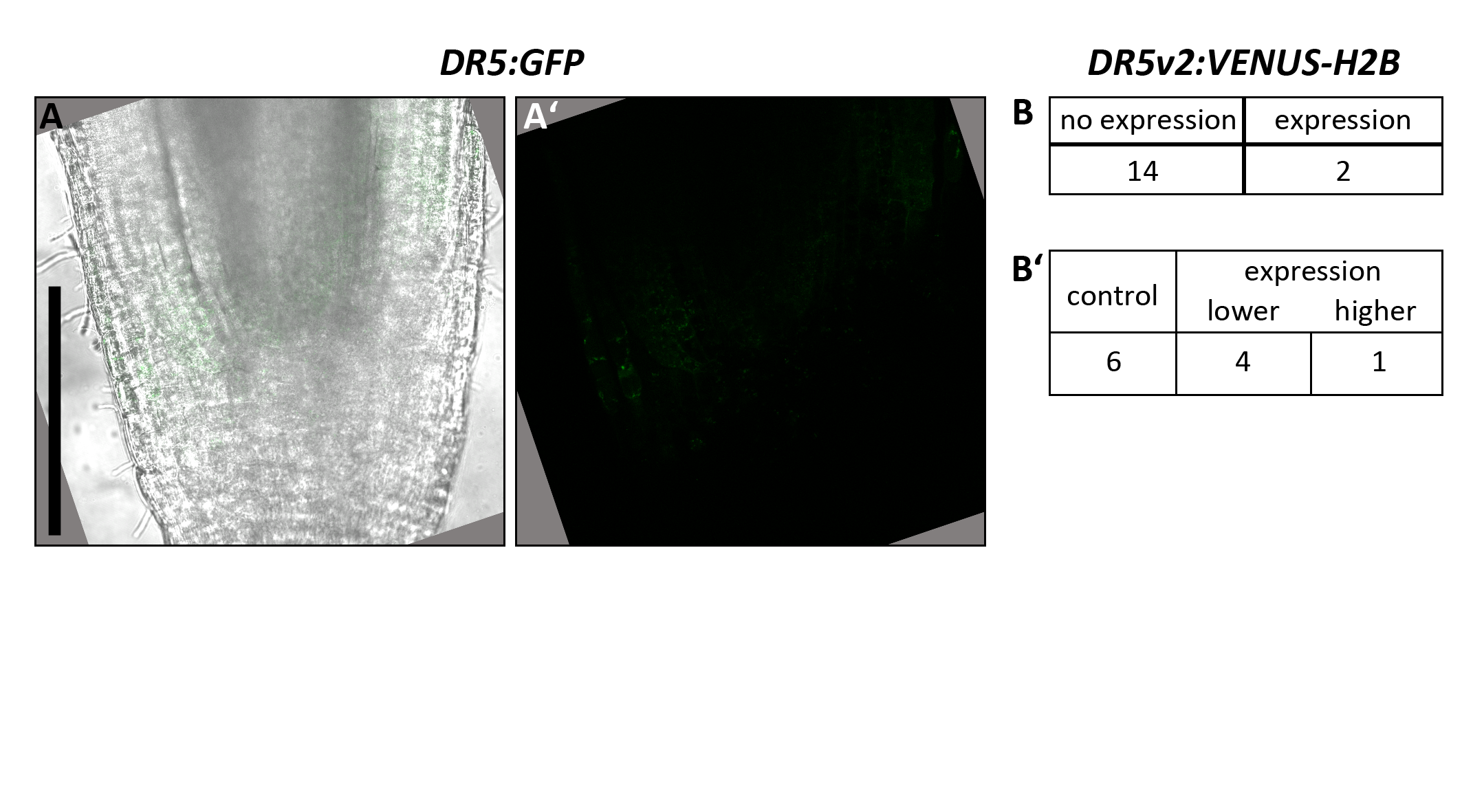

Supplement: S5 Fig — A) Exemplary picture of DR5:GFP root; transmitted light and (undetectable) GFP emission (A)), undetectable GFP emission only (A’)); four independent transgenic lines were examined and show no GFP epression; scale bar 200 μm. B) DR5v2:VENUS-H2B lines show only variable or no expression and do not show a consistent reaction on 2,4D treatment; number of expressing DR5v2:VENUS-H2B lines (B)); number of plants that show the respective expression change upon treatment with 10 μM 2, 4D for 24 h (B’)). (TIF) [file pone.0196086.s005.tif]

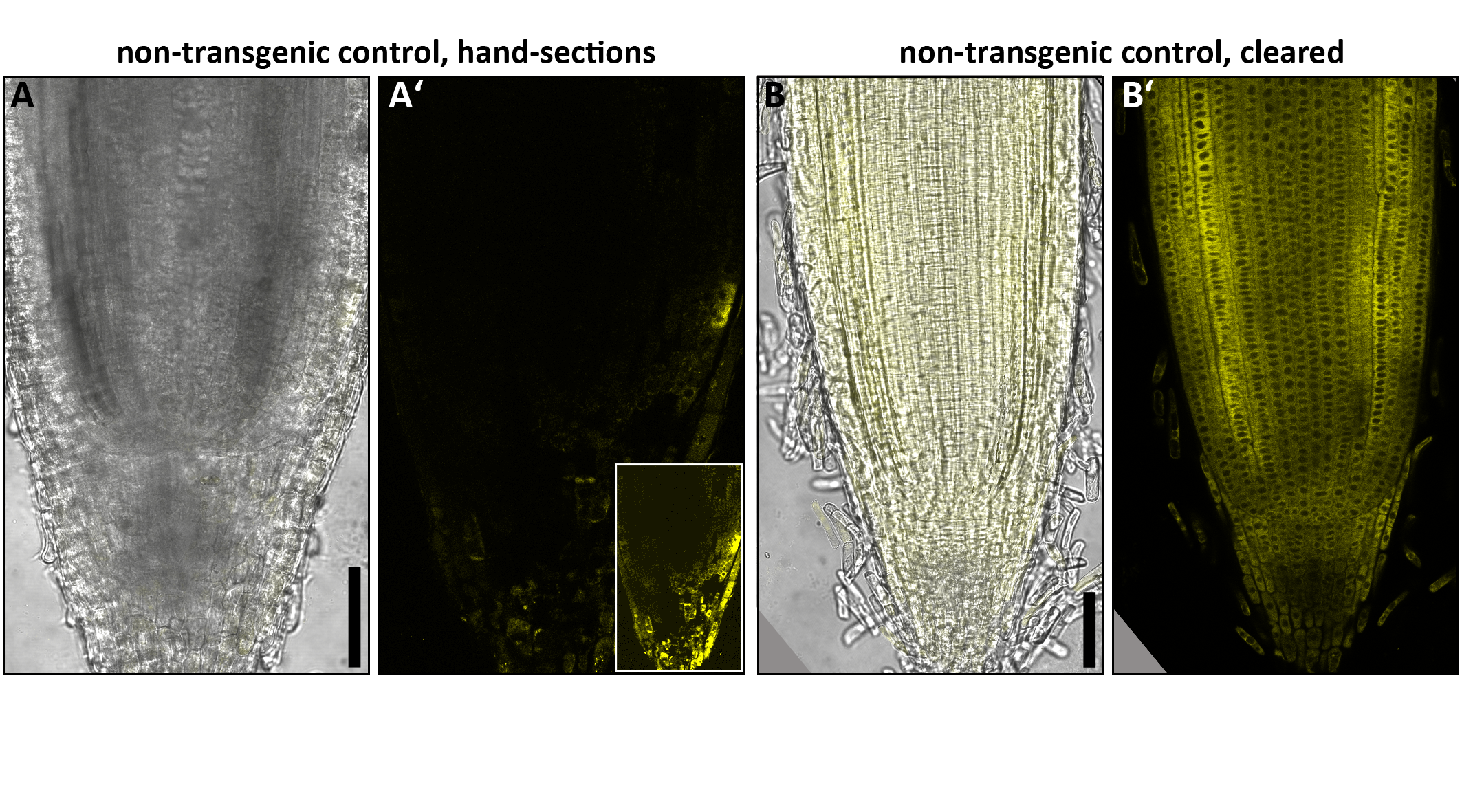

Supplement: S6 Fig — A) Representative picture of the root meristem of a non-transgenic Golden Promise seedling 8 DAG; transmitted light and mVENUS emission (A)), mVENUS emission only (A’)), same settings as in Fig 4B and 4B’; hand-sections as described in Material and Methods; only background signal with mVENUS excitation. B) Representative picture of the root meristem of a non-transgenic Golden Promise seedling 8 DAG; transmitted light and mVENUS emission (B)), mVENUS emission only (B’)), same settings as in Fig 5A’; cleared as described in Material and Methods; only background signal with mVENUS excitation; scale bars 100 μm; inset in A’) shows respective pictures with tonal correction to show autofluorescence. (TIF) [file pone.0196086.s006.tif]

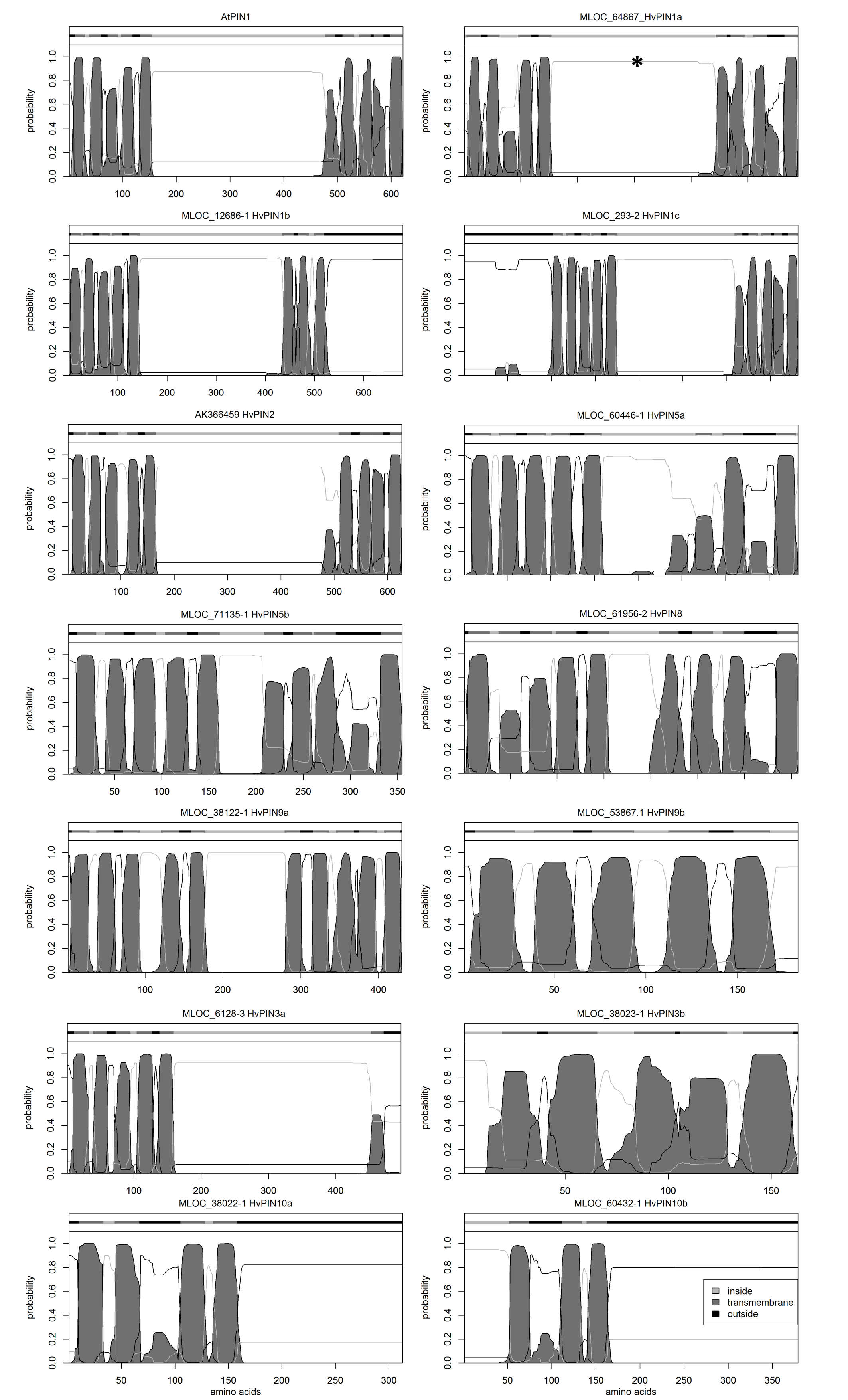

Supplement: S7 Fig — Topology of the transmembrane barley PIN proteins in comparison to AtPIN1; domains predicted to the inside of the cell are shown in light-gray, transmembrane domains are shown in dark-gray and domains outside the cell are depicted in black according to the legend; in the protein topology of MLOC_64867—HvPIN1a the asterisk marks the site where mVENUS is inserted for the reporter line shown in Fig 5; newly identified HvPINs are named according to their topology and the cluster of the Arabidopsis, maize and rice PIN family to which they belong. (TIF) [file pone.0196086.s007.tif]
